# Prevalence, motivation, and outcomes of clinic transfer in a clinical cohort of people living with HIV in North West Province, South Africa

**DOI:** 10.1186/s12913-022-08962-8

**Published:** 2022-12-26

**Authors:** Hannah H. Leslie, Alyssa C. Mooney, Hailey J. Gilmore, Emily Agnew, Jessica S. Grignon, Julia deKadt, Starley B. Shade, Mary Jane Ratlhagana, Jeri Sumitani, Scott Barnhart, Wayne T. Steward, Sheri A. Lippman

**Affiliations:** 1 Division of Prevention Science, Department of Medicine, University of California, San Francisco, 550 16th Street, San Francisco, CA 94158 USA; 2 Institute for Health Policy Studies, University of California, San Francisco, San Francisco, CA USA; 3 Department of Global Health, University of Washington, Seattle, WA USA; 4 International Training and Education Center for Health (I-TECH) South Africa, Pretoria, Republic of South Africa

**Keywords:** HIV, Clinic transfers, Mobility, Retention in care, South Africa

## Abstract

**Introduction:**

Continuity of care is an attribute of high-quality health systems and a necessary component of chronic disease management. Assessment of health information systems for HIV care in South Africa has identified substantial rates of clinic transfer, much of it undocumented. Understanding the reasons for changing sources of care and the implications for patient outcomes is important in informing policy responses.

**Methods:**

In this secondary analysis of the 2014 – 2016 I-Care trial, we examined self-reported changes in source of HIV care among a cohort of individuals living with HIV and in care in North West Province, South Africa. Individuals were enrolled in the study within 1 year of diagnosis; participants completed surveys at 6 and 12 months including items on sources of care. Clinical data were extracted from records at participants’ original clinic for 12 months following enrollment. We assessed frequency and reason for changing clinics and compared the demographics and care outcomes of those changing and not changing source of care.

**Results:**

Six hundred seventy-five (89.8%) of 752 study participants completed follow-up surveys with information on sources of HIV care; 101 (15%) reported receiving care at a different facility by month 12 of follow-up. The primary reason for changing was mobility (*N*=78, 77%). Those who changed clinics were more likely to be young adults, non-citizens, and pregnant at time of diagnosis. Self-reported clinic attendance and ART adherence did not differ based on changing clinics. Those on ART not changing clinics reported 0.66 visits more on average than were documented in clinic records.

**Conclusion:**

At least 1 in 6 participants in HIV care changed clinics within 2 years of diagnosis, mainly driven by mobility; while most appeared lost to follow-up based on records from the original clinic, self-reported visits and adherence were equivalent to those not changing clinics. Routine clinic visits could incorporate questions about care at other locations as well as potential relocation, particularly for younger, pregnant, and non-citizen patients, to support existing efforts to make HIV care records portable and facilitate continuity of care across clinics.

**Trial registration:**

The original trial was registered with ClinicalTrials.gov, NCT02417233, on 12 December 2014.

## Introduction

An estimated one-third of patients living with HIV will be reported as lost to follow-up in Sub-Saharan Africa within three years of initiating antiretroviral therapy (ART) [1]. Those recorded as lost include individuals permanently leaving care as well as those who discontinue and then re-initiate HIV care in different locations [2]. Patients may change clinics for multiple reasons, including relocation; seeking care closer to home, work, school, or family; seeking higher quality care; and, for HIV care in particular, avoiding discrimination or loss of confidentiality in health services, whether anticipated or experienced [3]. Active clinic selection, including bypassing nearby facilities and changing facilities during treatment, can reflect both the challenges of primary care systems that face substantial resource constraints and pervasive stigma [4, 5].

Existing data suggest changing sources of care for adults receiving HIV care and treatment is common but infrequently documented in official transfer records. A 2015 systematic review of studies across countries in sub-Saharan Africa found that 19% of those lost to follow-up at one facility had actually initiated care elsewhere [6]. In South Africa, home to the world’s largest HIV/AIDS epidemic and the world’s largest free ART program [7–11], an analysis linking patient records using national lab data revealed that 6-year retention in any care was 63%, much higher than the 29% retained at initiating clinic [12]. Recent detailed assessments of clinical records in government facilities in rural Mpumalanga Province have found that patient tracking overestimates loss to follow-up and underestimates clinic changes: nearly 1 in 3 patients recorded as lost to follow-up had initiated care elsewhere, with many transfers undocumented by the original facility [13, 14]. Studies in urban centers have also identified moderate rates of clinic change and more common treatment gaps or cessation among those changing their source of care [15, 16].

Identifying the motivating factors for changing clinics and the effects on continuity of care can inform intervention approaches: changes driven by population mobility can be best addressed through improved record systems to ensure continuous care, while changes driven by stigma or poor quality care require additional health system responses to improve services. Existing studies in South Africa are limited to clinical record review or qualitative assessment [14, 16–19]; few studies have been able to quantify reasons for changing source of care for chronic conditions such as HIV [20].

We assessed clinic transfer practices in an HIV-positive clinic-based cohort in South Africa [21, 22]. We examined the scope and reasons for self-reported clinic changes, including the role of mobility and of quality of health services, described individual characteristics of those changing and not changing sources of care, and compared clinical and self-reported care outcomes between these groups.

## Methods

### Study setting

This secondary analysis is based on the I-Care study, conducted in the Bojanala Platinum District of North West Province, South Africa [21, 22]. The district is home to 1.5 million people; mining and agriculture are the major sources of revenue, with 40% of economically active adults unemployed [23]. The prospect of mining employment draws largely male migrants into Bojanala from within South Africa (Eastern Cape) as well as from Mozambique, Lesotho, and eSwatini [24, 25]. Study enrollment occurred from October 2014 to April 2015 and follow-up continued through May 2016. At the time of the study, 1 in 5 adults in the province and nearly 1 in 3 pregnant women were living with HIV [22, 26, 27]. Bojanala District was not part of the 2014-2016 national pilot program for differentiated care interventions: HIV care was provided centrally within Department of Health primary care facilities [28]. Under national guidelines, individuals testing positive for HIV were eligible for treatment if pregnant or based on disease severity (CD4 count ≤350 at study start, CD4 count ≤500 as of January 2015, WHO clinical stage 3 or 4, or active tuberculosis or hepatitis B co-infection) [29].

### Data source

The I-Care study was a cluster-randomized trial to assess efficacy of SMS-messaging and peer navigation in improving linkage to and retention in HIV care. Study rationale, design, and clinic selection are detailed elsewhere [21, 22]. Participants were adults (≥18 years old) diagnosed with HIV within the past year at time of study enrollment in one of 17 public clinics serving the general population in Bojanala Platinum District [22]. Participants’ clinical data were extracted from the 17 participating clinics for the study period. Using computer-assisted personal interview (CAPI), interviewer-administered surveys were conducted at enrollment, 6 months, and 12 months in the most widely used local language, Setswana, at their clinic of care; in rare instances follow-up questionnaires were done by phone. Participants permanently relocating out of the area, while no longer able to receive study interventions, were still contacted to complete follow up surveys when possible. Individuals completing a follow-up survey at 6- and/or 12 months were eligible for this secondary analysis.

### Measures

Clinic change was defined as self-report of receiving HIV care at another clinic in time since diagnosis at six and/or twelve month follow up. While the measure specified “ever” receiving HIV care at another clinic, since study eligibility required diagnosis up to 1 year prior to enrollment and the question was posed up to 1 year post-enrollment, the change must have occurred within the past 2 years. Respondents reported transferring in (receiving care for <3 months or >3 months at another clinic before coming to the study clinic) and transferring out (going to another clinic permanently). Reasons for the change were provided as multiple choice options, including an option to specify other reasons, and collapsed into 3 categories: mobility (e.g. temporary absence, permanent relocation), quality of care (preferences related to the receiving facility, confidentiality concerns, other personal experiences), and referral to higher level care. The 12-month survey asked the name of the other clinic used; named facilities were mapped and classified as within North West Province, in a different province, or outside South Africa.

We defined self-reported measures of service use: number of visits for HIV care in the past 12 months and treatment adherence. Adherence was defined as reporting taking ART at least 29 of the past 30 days (95% adherence) among those on treatment. Responses from the 12-month survey were used unless respondents completed only the 6-month survey, in which case the recall period for clinic visits was also changed to 6 months. For comparison to self-reported data, we extracted number of visits recorded at study clinic in the 12 months prior to final interview from clinic documentation. From the main I-Care trial analysis, retention in care was based on clinic documentation and defined as having a care appointment at least every 94 days, given routine dispensation of 90-day medication supplies and the South African guidance that no more than 4 days of medication should be missed per 90 days to achieve >95% adherence [29]. Those not yet initiated on ART were defined as retained if they attended at least 2 visits in the past 12 months, within 2 months of semiannual CD4+ T-cell monitoring visit target dates.

Mobility was explored among all participants in the demographic portion of the survey by asking about changing residence and months away from home in the past 6 months as well as the primary reason for time away. Participants were also asked if they had visited another clinic specifically to confirm their HIV test result in the time since diagnosis. Additional demographic variables were extracted from baseline survey responses and categorized for analysis, including age (18-24, 25-29, 30-34, 35-39, 40-49, 50-65), education (primary or less, some secondary, completed secondary or higher), relationship status (married/cohabitating, partnered but not living together, single and never married, single and separated or widowed), pregnancy status at time of diagnosis, employment (full time, part time, self-employed/student/other, unemployed), and prior use of ART.

### Statistical analysis

We compared individuals eligible for analysis to the baseline study population on demographic variables and HIV care history using descriptive statistics and Chi-square tests (categorical variables) and Kruskal-Wallis tests (continuous variables). We used descriptive statistics to quantify prevalence of clinic changing and reasons for change. We then compared individuals reporting clinic change to those not remaining in study clinics by demographic variables to characterize those more likely to change facilities. In order to evaluate care and treatment outcomes including ART initiation and clinic attendance, adherence, and retention among those on ART, we compared self-reported and clinic data for these outcomes using non-parametric comparisons (Fisher’s exact test for categorical variables and Kruskal-Wallis tests for continuous variables) and adjusted multivariable regression. We used generalized estimating equation (GEE) models with a logit link for categorical outcomes and linear for continuous outcomes; we accounted for clustering by clinic and use exchangeable correlation structure with robust standard errors following the I-Care trial analytic approach. We controlled for gender and pregnancy at time of diagnosis (among women), age categories, South African citizenship or residency, and I-Care study arm. We compared self-report and clinic data for visits in the past 12 months using a paired t test. Analyses were conducted in Stata v17 (StataCorp, College Station, TX).

## Results

Of 752 individuals enrolled in the study, 89.8% (675) responded to surveys at 6 and/or 12 months, including 8.6% (65) responding only to the 6-month survey. As shown in Table 1, the 77 individuals excluded from analysis were more likely to be male (52% (30) of those without follow up vs. 39% (292) of all enrolled) and to be diagnosed with HIV within 1 month prior to enrollment (81% (62) vs. 65% (487) among all enrolled). They were less likely to be on ART at or before study enrollment (24% (18) vs. 35% (264) overall). Median age of those excluded was 32 years (IQR 26 – 39), younger but not significantly different from all participants (median 34 years, IQR 27 – 41). Those excluded from analysis due to not completing either follow-up survey were still tracked in clinical records; 29% (22 of 77) died during the study and 35% (21) had zero clinic visits during the study compared to 8% (56) of survey participants, suggesting many left study clinics as well as study participation.

**Table 1:** Baseline characteristics of I-Care participants based on availability of follow-up survey data

	Any follow-up	No follow-up	Total	
	(*N* = 675)	(*N* = 77)	(*N* = 752)	*p*-value
**Age (years)**				0.335
Median (Q1, Q3)	34.0 (27.0, 42.0)	32.5 (26.0, 39.0)	34.0 (27.0, 41.0)	
**Gender**				0.013
Male	252 (37.3%)	40 (51.9%)	292 (38.8%)	
Female	423 (62.7%)	37 (48.1%)	460 (61.2%)	
**Education**				0.066
Primary or less	122 (18.2%)	21 (28.0%)	143 (19.1%)	
Some secondary	301 (44.8%)	34 (45.3%)	335 (44.8%)	
Secondary or higher	249 (37.1%)	20 (26.7%)	269 (36.0%)	
**Relationship status**				0.472
Married/cohabitating	223 (33.0%)	31 (40.8%)	254 (33.8%)	
Partnered, not living together	177 (26.2%)	15 (19.7%)	192 (25.6%)	
Single, never married	228 (33.8%)	24 (31.6%)	252 (33.6%)	
Single, separated or widowed	47 (7.0%)	6 (7.9%)	53 (7.1%)	
**Pregnant at diagnosis**				0.543
No	266 (62.9%)	22 (57.9%)	288 (62.5%)	
Yes	157 (37.1%)	16 (42.1%)	173 (37.5%)	
**Employment**				0.524
Full time	163 (24.1%)	24 (31.6%)	187 (24.9%)	
Part time	82 (12.1%)	7 (9.2%)	89 (11.9%)	
Self-employed, student, other	79 (11.7%)	8 (10.5%)	87 (11.6%)	
Unemployed	351 (52.0%)	37 (48.7%)	388 (51.7%)	
**South African citizen or permanent resident**				0.096
No	94 (13.9%)	16 (21.1%)	110 (14.6%)	
Yes	581 (86.1%)	60 (78.9%)	641 (85.4%)	
**Past year location**				0.197
< 1 month away from home	540 (80.0%)	56 (73.7%)	596 (79.4%)	
≥ 1 month away from home	135 (20.0%)	20 (26.3%)	155 (20.6%)	
**Closest facility is usual source of care**				0.065
No	29 (4.3%)	0 (0.0%)	29 (3.9%)	
Yes	646 (95.7%)	76 (100.0%)	722 (96.1%)	
**Newly diagnosed (<1 month) with HIV**				0.002
No	250 (37.0%)	15 (19.5%)	265 (35.2%)	
Yes	425 (63.0%)	62 (80.5%)	487 (64.8%)	
**Ever on ART**				0.027
No	429 (63.6%)	58 (76.3%)	487 (64.8%)	
Yes	246 (36.4%)	18 (23.7%)	264 (35.2%)	
**Any visits to study clinic after enrollment**				
No	56 (8.3%)	27 (35.1%)	83 (11.0%)	<0.001
Yes	619 (91.7%)	50 (64.9%)	669 (89.0%)	

Participants eligible for analysis included 63% women (423) and 37% men (252), with a median age of 34; most had some secondary (45%, 301) or completed secondary education (37%, 249), and only 25% (163) were employed full time. Sixty-three percent (425) were diagnosed with HIV within 1 month prior to enrollment. Nearly all (96%, 646) of participants eligible for analysis sought care at the closest facility; the 4% (29) bypassing the nearest facility mentioned quality of care (45%), familiarity with the further facility (21%), and confidentiality (10%) as motivating reasons.

In follow-up surveys, a total of 101 participants (15%) reported receiving HIV care at a different clinic in the time since diagnosis (Table 2); of those receiving care elsewhere, 75% left the study clinic (76) – including 3 who previously transferred into the study clinic - while the rest reported seeking care elsewhere before enrolling or re-enrolling at the study clinic. Of 66 participants with known receiving clinics, 59% were within the province, including 4 of 11 non-citizens (36%) and 35 of 55 South African residents / citizens (64%).

**Table 2:** Frequency and reasons for changing clinics (*N*=101)

	N (%)
Clinic change (received HIV care at a different clinic in time since diagnosis)	
Into study clinic	25 (24.8)
Out of study clinic	76 (75.2)
Total	101 (15.0)
Reason for most recent change^a^	
Mobility	78 (77.2)
Poor quality of care	14 (13.9)
Referral	12 (11.9)
Location of receiving clinic^b^	
Within province	39 (59.1)
Outside province	21 (31.8)
Outside country	6 (9.1)

^a^Individuals reporting changing at both 6 and 12 months could provide a reason at each survey

^b^Data on receiving clinic available for 66 of those reporting clinic change

Mobility was the major driver of clinic change: 77% (78) reported mobility as the reason for changing. These included affirmative responses on reason for change item options such as “I moved,” “I was temporarily away from home/traveling,” and other-specify responses such as temporary relocation for employment or family responsibilities, including to deliver a child. No women reported a change in clinic specifically related to the end of antenatal care (ANC). Reasons related to quality of care were less common, reported by 14% of those changing. These included options of “I like this clinic more” (*N*=5), “I was worried about confidentiality” (*N*=1), and other-specify responses related to bad, crowded, slow, or costly care.

As compared to those who did not change clinics, individuals reporting change in source of care were younger (median age of 30 at baseline vs. 34), more likely to have been pregnant at diagnosis, and less likely to be South African citizens or permanent residents (Table 3). Gender, education, and employment did not differ. There were important differences in HIV history: individuals who changed clinics had been diagnosed more recently and were more likely to visit multiple clinics to confirm HIV test result.

**Table 3:** Characteristics of individuals changing vs. not changing HIV care facility in 2 years following diagnosis (*N*=675)

	No change (***N***=574)	Change (***N***=101)	
**Baseline measures**			
**Demographics**	**N (%) or Median (IQR)**		***P*-value**
Age	34.0 (27.0, 42.0)	30.0 (25.0, 37.0)	0.001
Gender			0.203
Male	220 (38.3%)	32 (31.7%)	
Female	354 (61.7%)	69 (68.3%)	
Education			0.449
Primary or less	106 (18.6%)	16 (15.8%)	
Some secondary	259 (45.4%)	42 (41.6%)	
Completed secondary or higher	206 (36.1%)	43 (42.6%)	
South African citizen or permanent resident	502 (87.5%)	79 (78.2%)	0.013
Pregnant at diagnosis			0.009
No	231 (65.6%)	35 (49.3%)	
Yes	121 (34.4%)	36 (50.7%)	
Employment			0.671
Full time	139 (24.2%)	24 (23.8%)	
Part time	73 (12.7%)	9 (8.9%)	
Self-employed, student, other	68 (11.8%)	11 (10.9%)	
Unemployed	294 (51.2%)	57 (56.4%)	
**Follow-up measures**			
**Location during study period**			
Moved or changed residence in past 6 months	91 (15.9%)	58 (57.4%)	<0.001
≥ 1 month away from home in past 6 months	83 (14.5%)	33 (32.7%)	<0.001
**HIV testing**			
Months since diagnosis	12.7 (12.0, 16.1)	12.3 (11.9, 13.9)	0.014
Visited multiple clinics to confirm HIV test result	28 (4.9%)	23 (22.8%)	<0.001

Those reporting change in clinic were more likely to report a change in residence (57% vs. 16%) or having spent at least 1 month away from home (33% vs. 15%) in the 6 months prior to follow up assessment. Among participants reporting at least 1 month away, duration away was longer for those changing source of care: 2.7 months vs. 2 months on average. The most common reason for being away among all respondents was to visit family or friends (47%, 45% of those changing clinics), while 22% (26) of all 116 participants with time away and 15% (5) of those changing clinics with time away reported employment-related reasons (data not shown).

Participants reported high ART initiation: 98% (99) of those changing clinics and 86% (496) among those not changing. The adjusted odds of reporting ART use were 7.7 times higher among those changing clinics than those remaining in study clinics (95% confidence interval (CI) 2.21 to 31.05, Table 4). Among those on ART, self-reported number of clinic visits (median 7 in the past 12 months) and ART adherence in the past 30 days (94%) did not significantly differ between those changing clinics and remaining.

**Table 4:** HIV care and treatment outcomes of individuals changing vs. not changing HIV care facility in 2 years following diagnosis (*N*=675)

	No change (***N***=574)	Change (***N***=101)		
	N (%) or Median (IQR)		Non-parametric comparison ***p*** value^b^	Adjusted model^c^
**Self-reported measures**				
Ever on ART	496 (86.4%)	99 (98.0%)	<0.001	AOR = 7.73 (1.97, 30.35)
Clinic visits past 12 months (ever ART, *N*=595)^a^	7 (6, 12)	7 (6, 11)	0.362	β = -0.47 (-1.20, 0.26)
>95% ART adherence in past 30 days	446 (93.7%)	88 (93.6%)	1.000	AOR = 0.98 (0.38, 2.52)
**Study clinic documentation**				
Ever on ART	505 (88.0%)	80 (79.2%)	0.025	AOR = 0.46 (0.22, 0.93)
Clinic visits past 12 months (ever ART, N=585)^a^	7 (5, 9)	4 (2, 6)	<0.001	β = -2.72 (-3.62, -1.82)
Retained in care (ever ART)	307 (60.8%)	9 (11.2%)	<0.001	AOR = 0.09 (0.03, 0.23)

^a^past 6 months if no 12-month survey

^b^Kruskal-Wallis test for continuous variables, Fisher’s exact test for categorical variables

^c^Controlling for age categories, gender and pregnancy at diagnosis, South African residency, and I-Care trial arm

The sub-analysis of ART clients remaining at study clinics - whose clinic records should be complete - showed that the average number of clinic visits was slightly higher on self-report than in study clinic documentation (difference = 0.66 visits, t test *p* < 0.01). Those changing clinics reported substantially more clinic visits than were recorded at the study clinic (difference = 3.81 visits, t test *p* < 0.01). Using study clinic records alone, ART initiation as well as ART clients’ attendance and retention were all significantly lower for those changing clinics than those remaining in adjusted models.

## Discussion

Our study of a clinic-based sample of HIV-positive adults in a rural area of North West Province, South Africa, indicated that 15% had received HIV care at multiple clinics within the first 2 years of diagnosis, primarily driven by population mobility. Younger individuals, those pregnant at diagnosis, and non-citizens were more likely to change clinics, characteristics that could be used to predict changes in routine care. Self-reported measures did not show reductions in ART initiation, clinic visits among those on ART, or ART adherence associated with changing clinics, although documentation at original clinics did show differences. A major implication of these findings is the need to track care outcomes at the individual level rather than relying only on clinic-based records to ensure appropriate monitoring of care engagement and outcomes in the setting of a mobile population [30, 31].

Prevalence of clinic transfer in this study (15% of those retained in the study) was comparable to studies in urban settings in South Africa that have found 8% incidence of transfer among general HIV patients and 13-21% transfer among postpartum women. Pregnant women were more likely to transfer care in our study as well, although none cited the end of ANC as the reason for a change in facility. This finding potentially reflects mobility among childbearing women and mothers of young children, including extended family visits [15, 18, 19]. The large majority of clinic changes in our data were driven by population mobility, most of which was within the province. Interestingly, our findings do not support the idea that people commonly “shop” for clinics as a primary reason for transfer, at least in this largely rural area. The belief that people seek care further from their home to avoid stigma and/or shop around for better care was brought up by local clinic personnel anecdotally during project data collection and has been reported in previous studies [32], but only 1 participant specifically cited confidentiality concerns as a reason to change and a handful noted other quality issues such as delays and crowding. Our data indicate that it is more likely that people appeared at multiple clinics for diagnosis confirmation and/or to re-engage after a move or while away from home.

Previous studies have found gaps in continuity of care among those electing to change clinics despite the fact that many assumed lost to follow-up do in fact continue care elsewhere [12–14, 18]. In our study, we are unable to assess self-reported outcomes for the individuals who did not respond to follow-up surveys and were not confirmed deceased; clinic records indicated low retention. Among those who responded to follow-up surveys, we found no evidence of ill effects of clinic transfer on ART initiation, treatment visits, or recent adherence based on self-reported measures, suggesting that those changing clinics successfully linked to care elsewhere. We were able to directly compare number of clinic visits between self-report and clinic documentation for those on ART reporting a single source of care: these figures were generally consistent, with self-report overestimating total clinic visits by less than 1 visit, potentially due to inexact recall and social desirability. If self-reported measures were similarly reliable among those changing clinics, our findings would suggest minimal impact on care outcomes. It is possible, however, that overreporting of clinic visits (and other outcomes) could be more pronounced among those changing clinics, for instance due to awareness that answers could no longer be compared to clinic records or enhanced desire to demonstrate normative behavior of adherence to care after reporting the potentially less desirable behavior of changing clinics [33].

Clinic-based measures would suggest those leaving study clinics had lower likelihood of ART initiation, fewer treatment visits, and much lower retention. These differences reflect at least in part the lack of documentation of care for those leaving study clinics without indication of a formal transfer; though official transfer letters were documented, they were rare. While our data do not enable a definitive assessment of adherence to care and treatment among those changing clinics, the fact that these participants were willing to participate in follow-up surveys and reported comparable care outcomes suggests that many patients were motivated to maintain care and that record linkages could be possible with increased patient tracing efforts.

This study took place from 2014 to 2016; multiple changes to National Department of Health in South Africa policy have since taken place, including scaling up of differentiated care models to decongest clinics [34], a universal test and treat policy taking effect in September 2016, and in 2019, the recommendation of Tenofovir, Lamivudine, and Dolutegravir (TLD) as the first-line regimen of choice [35]. Making ART universal, reducing wait times for those stable on ART, shifting pick up points to community settings, and instituting a simpler regimen with fewer side effects can all improve patient experience and contribute to decreased disengagement from care early in treatment. While the context of HIV treatment has evolved, our findings speak to the importance of recognizing individual mobility in shaping treatment trajectories. The need to develop models for accurate care engagement tracking remains present today. Some promise for tracking lies in the Synchronised National Communication in Health (SyNCH) system for tracking medication pick up in differentiated care models using unique national ID numbers and in South Africa’s National Health Insurance policy, which includes plans to register the population and issue to each member an insurance card using their unique identifier linked to the Department of Home Affairs [36–38]. Models for moving away from institution-centered record systems in South Africa to internet-based personal health records to consolidate and coordinate lifelong health information have been proposed but not yet adopted [39, 40].

In the interim, our data suggest that challenges for continuity of care were compounded primarily by high mobility rather than poor quality of care or stigma concerns in this population. Our sample indicated frequent moves, a large proportion of which was for extended visits to family and friends, reflecting the fluidity of “home,” the temporary nature of employment opportunities, and circular migratory patterns found in previous research in South Africa [41, 42]. Other studies indicate that patients with official transfer letters tend to rapidly re-engage [2]; while we did not observe these being implemented frequently at our study sites, encouraging their use could enhance retention post-transfer in this population. To that end, incorporating questions about upcoming travel for work or visits to family/friends during clinic visits could provide opportunities for clinicians to formally facilitate these transfers to reduce delays in re-engagement, improve patient outcomes, and coordinate information exchange between clinics. Such assessment may be particularly important for younger patients, pregnant women, and non-citizens based on our findings. Furthermore, questions at care initiation regarding previous clinics attended may also aid tracking retrospectively. Given the importance of mobility rather than confidentiality concerns reported in this sample, it is possible these findings are relevant to chronic health conditions beyond HIV.

That said, previous research has found that mobility-related barriers to engagement in care and adherence are often structural, such as stigma and fear of disclosure at the destination, and the inability to obtain sufficient medication supplies during travel [43]. Mobility can also catalyze a chain of events that lead to poor retention: a move may occur in the context of economic and social challenges, which intersects with other barriers to care, such as a lack of social support, a fear of harsh treatment from healthcare workers if visits were missed, and ART side effects exacerbated by food insecurity [44, 45]. Strategies to address these challenges as a component of care planning, such as through coordination and integration with other care support programs, would likely help to ensure continuity of care tailored to the unique motivations for and patterns of mobility.

These findings are based on a cohort with both clinical and self-reported data, providing insight on patient decisions in the time following HIV diagnosis. Our study was subject to limitations: clinic record tracking was incomplete specifically for those leaving study clinics, and while we tried to capture documentation of formal transfers from clinic files, few such records were found. Without documentation from all receiving clinics, we are unable to assess if self-reported measures may be differentially biased among those reporting clinic change. Information on destination clinic was not available for one third of those changing sources of care.

## Conclusions

The frequency of clinic transfer, neutral reasons for change, and reported re-engagement in care among participants in this study in rural North West Province suggest that research and policy should address identification of patients likely to move and documentation of transfers in sending and receiving clinics to reduce gaps in care and ensure efforts to find and re-engage patients are focused on those truly disengaged from care.

## Data Availability

The datasets used and/or analysed during the current study are available from the corresponding author on reasonable request.
